# Development and validation of PCR marker array for molecular selection towards spring, vernalization-independent and winter, vernalization-responsive ecotypes of white lupin (*Lupinus albus* L.)

**DOI:** 10.1038/s41598-025-86482-1

**Published:** 2025-01-21

**Authors:** Anna Surma, Michał Książkiewicz, Wojciech Bielski, Bartosz Kozak, Renata Galek, Sandra Rychel-Bielska

**Affiliations:** 1 Department of Gene Structure and Function, Institute of Plant Genetics, Polish Academy of Sciences, Strzeszyńska 34, 60-479 Poznań, Poland; 2 Department of Genetics and Plant Breeding, Poznań University of Life Sciences, Dojazd 11, 60-631 Poznań, Poland; 3 Department of Genetics, Plant Breeding and Seed Production, Wrocław University of Environmental and Life Sciences, Plac Grunwaldzki 24A, 50-363 Wrocław, Poland

**Keywords:** Flowering, Vernalization, White lupin, Marker-assisted selection, Spring sowing, Winter sowing, Plant genetics, Plant breeding

## Abstract

White lupin (*Lupinus albus* L.) is an ancient grain legume that is still undergoing improvement of domestication traits, including vernalization-responsiveness, providing frost tolerance and preventing winter flowering in autumn-sowing agriculture, and vernalization-independence, conferring drought escape by rapid flowering in spring-sowing. A recent genome-wide association study highlighted several loci significantly associated with the most contrasting phenotypes, including deletions in the promoter of the *FLOWERING LOCUS T* homolog, *LalbFTc1*, and some DArT-seq/silicoDArT loci. The present study aimed to develop and validate a versatile PCR marker array enabling molecular selection of spring- and winter-type white lupin ecotypes. Candidate DArT-seq and silicoDArT loci were transformed into cleaved amplified polymorphic sequence (CAPS) or derived CAPS markers. Developed markers, together with those previously published for *LalbFTc1* INDELs and quantitative trait loci from linkage maps, were implemented for screening of white lupin germplasm panel subjected to 2-year phenotyping of phenology traits. Three DArT-seq, two silicoDArT and seven *LalbFTc1* INDEL markers were positively validated, constituting a convenient PCR-based marker assay for rapid and accurate reselection of white lupin germplasm towards early flowering and thermoneutrality or late flowering and vernalization-responsiveness, as well as for tracking high genetic and phenotypic diversity within white lupin landraces, revealed in the present study.

## Introduction

White lupin (*Lupinus albus* L.) is a grain legume crop cultivated in Greece and Egypt since ancient times^1–3^. During the last centuries, this species has been domesticated and distributed into numerous countries including, among others, Italy, France, Spain, Germany, Switzerland, Poland, Ethiopia and Australia^4–9^. White lupin has recently been recognized as a valuable nutritional replacement for soybean protein source in livestock feeding (mostly pigs and broilers) and in aquaculture (salmonids, cyprinids and perciform fish)^10–15^. High protein content, moderate oil content with a desirable ratio of omega-6 to omega-3 acids as well as other specific components, such as oligosaccharides, antioxidants and non-starch carbohydrates, make white lupin also an attractive component in human nutrition^16^. White lupin requires less mineral fertilization than many other crops due to symbiotic nitrogen fixation and physiological adaptations to phosphorus deficiency involving proteoid root development^17,18^. Moreover, in rotation farming, residual nitrogen can be partially recovered by succeeding crops such as wheat (*Triticum aestivum* L.) and oilseed rape (*Brassica napus* L.)^19^.

Despite the relatively long history of cultivation, white lupin requires significant improvement to become a competitive crop for temperate climate agroecosystems. The major breeding efforts have been focusing lately on anthracnose resistance and low alkaloid content with significant progress achieved in deciphering the molecular components underlying these traits^7–9,20,21^. Moreover, sequence-specific markers were provided to assist molecular selection for low alkaloid content and anthracnose resistance in breeding programs^7,8,20,22^.

Numerous accessions of white lupin, as many other temperate plant species, are responsive to vernalization during vegetative growth phase^23,24^. During domestication of the species, two ecotypes were selected by breeders: a winter-type, which requires at least 2 weeks of vernalization with temperatures about 1–6 °C to induce flowering, and a spring-type, with much lower vernalization requirements, including even thermoneutral accessions^23,25–29^. As vernalization responsiveness is positively correlated with frost tolerance during winter, it is the fundamental trait underlying white lupin adaptation to autumn sowing^28,30,31^.

Thus, the domestication process of the species in the Mediterranean temperate climate selected winter ecotypes and autumn sowing since antiquity, as these were adapted to the winter frost events and vernalization, utilizing better the late winter and early spring humidity and precipitation rates of the particular temperate climate. Breeders have recognized these traits and driven their selection for further improvement of white lupin in ‘60 s-70s^32,33^. Autumn sowing is carried out in warm cropping areas such as the Mediterranean basin, Western Europe and Australia, where white lupin was imported in the late 60s^34^. In contrast, in colder regions of temperate climate, which include central and eastern Europe, Russia, the Northern United States and southeastern Canada, spring sowing is preferred^4,5,35–37^. Vernalization responsiveness is disadvantageous in spring types because it unnecessarily delays crop flowering in the absence of low temperature after sowing and interferes with a drought escape strategy based on early phenology^38^.

Recent years have witnessed unprecedented advances in molecular resources for numerous plant species, including white lupin. Such resources developed for this species include, among others, a high-density linkage map carrying sequence-based markers originating from a recombinant inbred line (RIL) mapping population descending from a cross between the early flowering vernalization-independent Ukrainian cultivar Kiev Mutant and the late flowering vernalization-responsive Ethiopian landrace P27174, two reference genome sequences, and a pangenome assembly for 40 accessions representing cultivars and landraces including Kiev Mutant and P27174^7,39–41^. Quantitative trait loci (QTL) mapping in the Kiev Mutant × P27174 RIL population revealed the presence of several QTLs for flowering date related to vernalization responsiveness^7^. PCR-based markers anchored in those QTLs were developed and implemented for correlation analysis in a germplasm panel carrying both wild and domesticated accessions, highlighting significant marker-trait associations^42^. Nevertheless, for the most contrasting phenotypes (i.e. very early or very late) no single marker applicable for molecular selection was identified, probably due to different genetic backgrounds in those accessions and parental lines of the mapping population. To address this issue, a white lupin diversity panel^43^ carrying genotypes originating from three main climate zones (tropical, subtropical and temperate)^44,45^ was subjected to genome-wide association study (GWAS) of flowering time in controlled conditions (greenhouse without vernalization) and in three environments differing by intensity of vernalization, i.e. Mediterranean and subcontinental climate regions in Italy (autumn sowing) and suboceanic climate in France (spring sowing). GWAS revealed several loci significantly associated with flowering time, including newly identified deletions in the promoter region of the *FLOWERING LOCUS T* homolog, *LalbFTc1* gene (*Lalb_Chr14g0364281*), and DArT-seq/silicoDArT markers matching previously published QTL regions and designating a few novel loci on other chromosomes^46^. That study provided also several candidate DArT-seq/silicoDArT loci associated with the most contrasting phenotypes, awaiting implementation into a PCR array.

Therefore, the present study aimed to the development a versatile PCR array for marker-assisted selection of white lupin towards spring- and winter ecotypes, including rare alleles correlated with very early or very late flowering. Single nucleotide polymorphisms (SNPs, DArT-seq markers) from recent GWAS study^46^ were directly transformed into PCR markers using cleaved amplified polymorphic sequence (CAPS)^47^ or derived CAPS (dCAPS)^48^ approaches, whereas presence/absence variants (silicoDArT markers) were sequenced using flanking primers and eventually transformed into allele-specific PCR or CAPS/dCAPS markers. Newly developed PCR markers together with those previously published for white lupin flowering time QTLs^42,49^ and *LalbFTc1* gene promoter INDELs^46^ were validated in the set of 300 white lupin accessions and confronted with observations of plant phenology (days from sowing to floral bud emergence, start of flowering and end of flowering) from a 2-year controlled environment study.

## Results

### Controlled environment phenotyping highlights large variability of white lupin phenology traits

300 white lupin lines originating from several geographical localizations representative of different climatic conditions (Supplementary Table S1) were phenotyped in a greenhouse under ambient long-day photoperiod for plant phenology traits, i.e. the number of days from sowing to floral bud emergence, the start of flowering and end of flowering. To highlight differences in plant phenology between winter and spring ecotypes, neither pre-sowing nor post-sowing vernalization was applied. The mean number of days from sowing to floral bud emergence reached 57.9 ± 15.3 in 2020 year and 54.0 ± 16.1 in 2021, ranging from 36.0 ± 0.0 to 111.7 ± 0.9 days and from 33.0 ± 0.0 to 114.7 ± 3.1 days, respectively. The beginning of flowering (i.e. development of the first fully colored petal) was observed about two weeks after floral bud emergence, namely at 72.0 ± 16.5 days from sowing in the first year of the study and 69.1 ± 16.3 in the second year. The range of observed values spanned from 41.3 ± 0.5 to 127.0 ± 1.4 in 2020 year and from 40.7 ± 0.5 to 121.0 ± 0.0 in 2021. The end of flowering on the main inflorescence occurred about 10 days after flowering initiation, thus at 82.9 ± 14.1 days from sowing in 2020 year and 79.0 ± 15.3 in 2021, with the range of observed values from 62.0 ± 0.0 to 129 ± 0.0 and from 54.0 ± 1.4 to 128.0 ± 0.0, respectively. As the experiment was performed in a controlled environment, very high correlation coefficients between years were observed, reaching 0.97 for days from sowing to floral bud emergence, 0.96 for start of flowering and 0.95 for end of flowering (P-values 6.5 × 10^–177^, 2.4 × 10^–62^, and 4.3 × 10^–156^, respectively). A slightly decreasing value of the correlation coefficient (albeit still very high) during the progress of phenology phases may reflect the increasing influence of the environment during plant development. Observation data for studied traits and replicates are provided in Supplementary Table S2 whereas calculated mean values and standard deviation values are in Supplementary Table S3.

### Heritability

A consistent genetic influence on the expression of white lupin phenology traits was observed in both greenhouse experiments, conducted in 2020 and 2021. Namely, for the year 2020, the heritability values for days from sowing to the floral bud formation, the start of flowering, and the end of flowering were estimated at 0.41, indicating a moderate genetic contribution to the phenotypic variance in these traits. Similarly, in 2021, the heritability values for these traits were uniformly estimated at 0.40. These findings suggest a stable genetic effect on flowering traits across years, which emphasizes the potential for breeding programs to enhance these characteristics in white lupin by artificial selection towards desired phenology.

### Transformation of DArT-seq and silicoDArT loci into PCR markers enables precise selection of white lupin winter ecotypes

The set of 11 loci from the recent genome-wide association study^46^ was subjected to transformation into PCR-based markers. This set included five DArT-seq (Chr06_14434379, Chr08_12044717, Chr11_14834409, Chr13_1469866 and Chr16_572706) and six silicoDArT (Chr02_2625564_D, Chr07_16560064_D, Chr08_3090075_D, Chr11_5890565_D, Chr13_12561729_D, Chr25_4002891_D) loci (Supplementary Table S4). All DArT-seq loci were successfully transformed into CAPS^47^ markers using flanking PCR primers and commercially available enzymes recognizing target SNP sites. Observed agarose gel patterns of enzyme cleavage products matched those calculated in silico for both allelic phases^50,51^.

The procedure was more complex for silicoDArT loci, which required resequencing and search for candidate loci that could eventually underlie observed polymorphism. Such an approach yielded one allele-specific PCR (Chr13_12561729_D_PCR), one CAPS (Chr07_16560064_D_CAPS) and three derived CAPS (dCAPS)^48^ markers (Chr02_2625564_D_dCAPS, Chr08_3090075_D_dCAPS and Chr11_5890565_D_dCAPS). Due to the lack of DNA sequence polymorphism in the resequenced region, one silicoDArT locus, Chr25_4002891_D, remains unsolved. Besides expected products, two markers, Chr11_5890565_D_dCAPS and Chr11_14834409_CAPS, yielded additional rare absence alleles. As CAPS and dCAPS markers show codominant appearance, we were able to mark off heterozygotes. Nevertheless, heterozygosity level observed in this white lupin germplasm panel was rather low and ranged from 0–1.0% (markers Chr02_2625564_D_dCAPS, Chr08_12044717_CAPS, Chr11_5890565_D_dCAPS, Chr11_14834409_CAPS and Chr13_12561729_D_PCR) through 2.3–5.7% (markers Chr06_14434379_CAPS, Chr13_1469866_CAPS, Chr07_16560064_D_CAPS and Chr08_3090075_D_dCAPS) to 9.3% (marker Chr16_572706_CAPS). Heterozygotes usually had an intermediate phenotype as compared to lines carrying the opposite alleles in a homozygous state. The list of positively validated markers including selected restriction enzymes, the lengths of expected restriction products and allele count observed in the white lupin germplasm panel is provided in Table 1. Results of white lupin germplasm genotyping with DArT-seq and silicoDArT PCR-based markers are provided in Supplementary Table S5. Marker sequences were deposited in the public repository Zenodo under DOI https://doi.org/10.5281/zenodo.10689061.

**Table 1:** Newly designed PCR-based markers for DArT-seq and silicoDArT loci from recent genome-wide association study^46^ and allele count observed in white lupin germplasm panel.

Marker name	Enzyme	Reference allele products (bp)	Alternative allele products (bp)	Reference allele count	Heterozygote count	Alternative allele count
Chr02_2625564_D_dCAPS	*Hpa*II	25, 113	138	298	0	2
Chr06_14434379_CAPS	*Hpa*II	558	254, 334	277	7	16
Chr07_16560064_D_CAPS	*Hpy*CH4IV	14, 478	14, 179, 299	165	18	117
Chr08_3090075_D_dCAPS	*Nla*IV	21, 202	223	129	17	153
Chr08_12044717_CAPS	*Acu*I	181	77, 104	296	0	4
Chr11_5890565_D_dCAPS	*Mae*II	7, 134	7, 18, 116	295	0	5
Chr11_14834409_CAPS	*Aci*I	90, 91	181	293	2	5
Chr13_12561729_D_PCR	–	–	129	131	3	166
Chr13_1469866_CAPS	*Rsa*I	127, 333	127, 132, 201	270	13	17
Chr16_572706_CAPS	*Hae*III	35, 128	163	219	28	53

As the set of white lupin lines used in this study majorly overlapped with those used for GWAS study^46^ we were able to compare segregation patterns between original DArT-seq or silicoDArT loci and their corresponding PCR-based markers. Observed patterns were identical for Chr06_14434379_CAPS and Chr08_12044717_CAPS markers, as well as very similar (more than 96% identical scores) for Chr11_14834409_CAPS, Chr13_1469866_CAPS and Chr16_572706_CAPS markers. All of those markers were based on DArT-seq loci. SilicoDArT loci revealed much lower similarity between original and PCR-based scoring, revealing 87% identity for Chr11_5890565_D_dCAPS, 77% for Chr13_12561729_D_PCR, 67% for Chr07_16560064_D_CAPS, whereas below 50% for Chr02_2625564_D_dCAPS and Chr08_3090075_D_dCAPS markers.

**Fig. 1:**
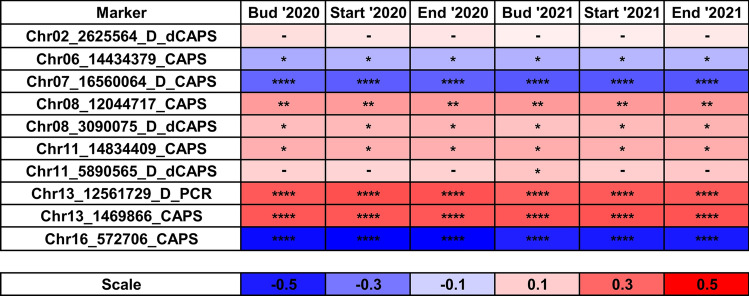
Correlation heatmap reporting Spearman rank correlation coefficients for each trait vs DArT-seq and silicoDArT PCR-based marker comparison. Reference alleles were coded as 0, heterozygotes as 1, whereas alternative alleles as 2. Observations were performed during 2020 and 2021 growing seasons in a greenhouse at the Institute of Plant Genetics, Polish Academy of Sciences, Poznań, Poland (52°26′ N 16°54′ E). The bar below the heatmap indicates the color legend of correlation coefficients. Asterisk (*) indicates significant correlations in the following scheme: ****, p < 0.00001; ***, 0.00001 ≤ p < 0.0001; **, 0.0001 ≤ p ≤ 0.001; *, 0.001 ≤ p ≤ 0.05.; –, non-significant.

All PCR-based markers anchored in DArT-seq and silicoDArT loci except the Chr02_2625564_D_dCAPS and Chr11_5890565_D_dCAPS markers revealed statistically significant correlations between marker scores and plant phenotype for all analyzed traits in both years. The direction of the relationship between genotype and phenotype was always the same among years and traits for a particular marker. The strongest correlations were observed for Chr16_572706_CAPS (mean r value − 0.53, P-value 5.6 × 10^–23^), Chr07_16560064_D_CAPS (mean r value − 0.36, P-value 1.4 × 10^–10^), Chr13_1469866_CAPS (mean r value 0.33, P-value 3.2 × 10^–9^) and Chr13_12561729_D_PCR (mean r value 0.33, P-value 5.5 × 10^–9^) markers. The Chr02_2625564_D_dCAPS marker turned out to be selective only to two Ethiopian genotypes (minor allele frequency, MAF, 0.7%), whereas the Chr08_12044717_CAPS marker (mean r value 0.20, P-value 5.7 × 10^–4^) just to four very late flowering genotypes representing French winter-type cultivars Luxe and Aster (MAF 1.3%). Similarly, the Chr11_14834409_CAPS marker (mean r value 0.16, P-value 0.006) was also selective to French winter-type cultivars (Adam and Aster), however, with a 1.0% false-positive calls, including two heterozygotes. The Chr13_1469866_CAPS marker was revealed to be fairly selective for late flowering non-domesticated germplasm from Azores and Canaries, which may be beneficial in breeding programs involving those landraces. Visualization of correlation coefficients for particular marker-trait associations is provided in Fig. 1.

### White lupin spring ecotypes can be efficiently selected with PCR markers anchored in *LalbFTc1* gene INDELs

PCR array of recently published 17 markers (Supplementary Table S4) tagging *LalbFTc1* gene promoter INDELs^46^ was used for white lupin genotyping to supplement the set of markers developed in the present study for several DArT-seq and silicoDArT loci. MAF ranged from 3.3% to 29.7%, with five markers with MAF below 10% (PR_36a–3.3%, PR_35a–5%, PR_58b–6.3%, PR_71b–7.7% and PR_42a–8.2%). Due to the dominant type of most of the *LalbFTc1* gene INDEL markers, heterozygotes were scored only for 5 markers. The level of heterozygosity ranged from 0.3% (PR_42a) to 7.0% (PR_58a). Comparing mean values, heterozygous lines for markers PR_35a and PR_71a conferred an intermediate phenotype as compared to lines carrying the opposite alleles in a homozygous state, whereas PR_58a marker heterozygotes were earlier than the corresponding homozygous lines. The list of *LalbFTc1* gene promoter INDEL markers including the lengths of products for reference and alternative alleles and allele count observed in white lupin germplasm panel is provided in Table 2. Results of white lupin germplasm genotyping with *LalbFTc1* gene promoter INDEL markers are provided in Supplementary Table S6.

**Table 2:** Markers used for PCR-based genotyping of INDEL polymorphism in the *LalbFTc1* gene (*Lalb_Chr14g0364281*) promoter^46^ and allele count observed in white lupin germplasm panel.

Name	Reference allele	Alternative allele	Reference allele count	Heterozygote count	Alternative allele count
PR_30	812 bp	no product	258	0	42
PR_35a	649 bp, no product	625 bp	280	10	10
PR_35b	649 bp	625 bp, heterozygote or no product	270	0	30
PR_36a	726 bp or heterozygote	482 bp or no product	290	0	10
PR_36b	726 bp or no product	482 bp or heterozygote	83	0	217
PR_39	no product	647 bp	240	0	60
PR_41	no product	766 bp	220	0	80
PR_42a	802 or 809 bp	774 or 781 bp	275	1	24
PR_42b	other variants	~ 850 bp	264	0	36
PR_58a	2504 bp	378 bp	247	21	32
PR_58b	2504 or 378 bp	no product or 116 bp	281	0	19
PR_58c	2504 or 378 bp or no product	116 bp	72	0	228
PR_70	no product	276 bp	240	0	60
PR_71a	222 or 229 or 250 bp	257 bp	244	10	46
PR_71b	250 or 257 bp	222 or 229 bp	275	4	21
PR_71c	other variants	~ 280 bp	266	0	34
PR_71d	250 bp	other variants	211	0	89

**Fig. 2:**
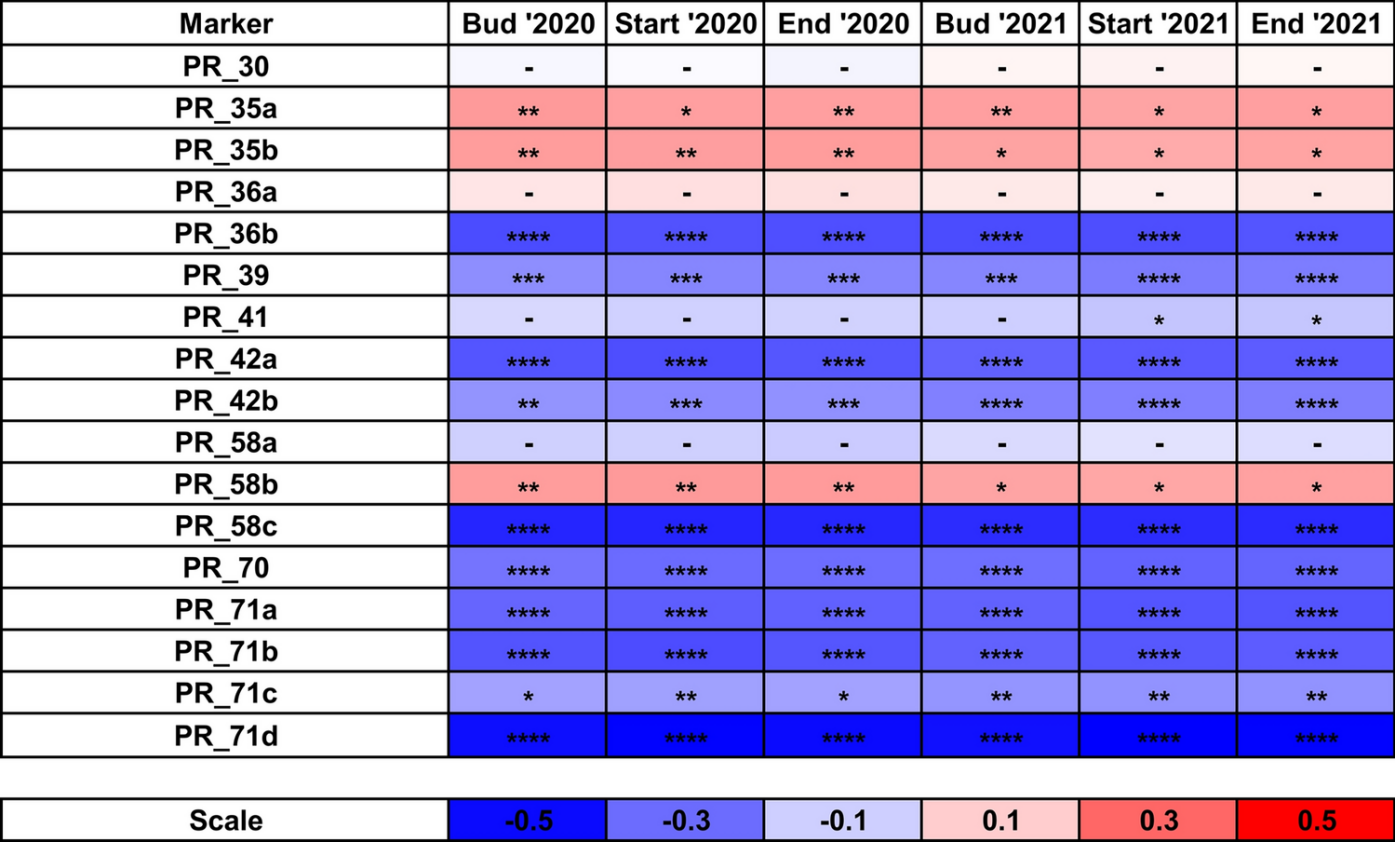
Correlation heatmap reporting Spearman rank correlation coefficients for each trait vs *LalbFTc1* gene INDEL PCR marker comparison. Reference alleles were coded as 0, heterozygotes as 1, whereas alternative alleles as 2. Observations were performed during 2020 and 2021 growing seasons in a greenhouse at the Institute of Plant Genetics, Polish Academy of Sciences, Poznań, Poland (52°26′ N 16°54′ E). The bar below the heatmap indicates the color legend of correlation coefficients. Asterisk (*) indicates significant correlations in the following scheme: ****, p < 0.00001; ***, 0.00001 ≤ p < 0.0001; **, 0.0001 ≤ p ≤ 0.001; *, 0.001 ≤ p ≤ 0.05.; –, non-significant.

All *LalbFTc1* gene promoter INDEL markers except PR_30, PR_36a and PR_41 revealed a statistically significant correlation between marker genotype and plant phenology (Fig. 2). The direction of the relationship was the same for a particular marker in both years. The strongest correlations were observed for markers PR_71d (mean r value − 0.51, P-value 5.6 × 10^–21^), PR_58c (mean r value − 0.44, P-value 1.5 × 10^–15^), PR_36b (mean r value − 0.36, P-value 1.4 × 10^–10^), PR_71b (mean r value − 0.34, P-value 1.2 × 10^–9^), PR_42a (mean r value − 0.34, P-value 1.3 × 10^–9^), PR_71a (mean r value − 0.33, P-value 4.5 × 10^–9^) and PR_70 (mean r value − 0.30, P-value 9.6 × 10^–8^). As all those markers had the same sign of the correlation coefficient (negative) but differed by a proportion of reference and alternative alleles, they provide a possibility to select the set of about 20 earliest white lupin lines (markers PR_42a and PR_71b ) or direct towards late flowering germplasm carrying about 70-80 white lupin lines (PR_36b and PR_58c). Together with the set of PCR-based markers anchored in DArT-seq and silicoDArT loci (Chr16_572706_CAPS, Chr08_12044717_CAPS and Chr11_14834409_CAPS), they constitute a convenient molecular tool to select with lupin germplasm resources expressing the most contrasting phenotypes of plant phenology.

### QTL markers from the white lupin linkage map provide lower selection efficiency than DArT-seq, silicoDArT and *LalbFTc1* gene INDEL markers

White lupin linkage mapping studies reported hitherto several QTLs associated with flowering time in this species^7,52^, that were recently supplemented with PCR-based markers enabling their tracking in white lupin germplasm^42,49^. In the present study, we analyzed the polymorphism pattern of 15 QTL markers originating from those studies (Supplementary Table S4). Screening of the white lupin germplasm panel revealed that only three QTL markers had MAF values below 15%, namely QTL15 (0.3%), QTL12 (1.5%) and QTL09 (2.5%). As all those markers appeared as codominant, we were able to distinguish heterozygotes from homozygotes. The heterozygosity level for this set of markers was remarkably higher than in two other marker systems reported in the present study, reaching a mean value of 6.1%, with a range from 0.0% (QTL15) and 0.3% (QTL11) to 13.7% (QTL12) and 14.0% (QTL10). The list of QTL markers including the lengths of products for reference and alternative alleles and allele count observed in the white lupin germplasm panel is provided in Table 3. Results of white lupin germplasm genotyping with QTL markers are provided in Supplementary Table S7.

**Table 3:** PCR-based markers for flowering time based on linkage mapping studies^7,42,49^ and allele count observed in white lupin germplasm panel.

Marker name	Polymorphism detection	Reference allele products (pb)	Alternative allele products (bp)	Reference allele count	Heterozygote count	Alternative allele count
QTL01 (MFT-FT3-F1)	Product length	295	311	231	22	47
QTL02 (FTc1-F4)	Product length	298	291	76	8	216
QTL03 (FY-F6)	CAPS, *Tsp*45I	233, 202	435	40	15	245
QTL05 (VIP3-F2)	CAPS, *BspL*I	114, 85, 70	184, 85	45	21	234
QTL06 (TP23903)	CAPS, *Bse*GI	64	49, 15	121	25	143
QTL07 (TP235608)	CAPS, *Afl*II	217	179, 38	168	26	106
QTL08 (TP94353)	CAPS, *Rsa*I	60, 51	111	74	22	204
QTL09 (SKIP1-F2)	dCAPS, *Bse*DI	79	48, 31	291	3	6
QTL10 (TP402859)	CAPS, *Hpa*II	198	112,86	78	42	180
QTL11 (FTa1-F2)	Product length	2218	1535	295	1	4
QTL12 (SEP3-F1)	dCAPS, *Taq*I	122, 23	145	146	41	113
QTL13 (TP86766)	CAPS, *Dde*I	64	48, 16	125	9	166
QTL14 (PIF4-F6)	CAPS, *Hpy*188III	138, 52	102, 52, 36	204	17	79
QTL15 (TP47110)	CAPS, *Hpy*F3I	42, 24	66	299	0	1
QTL16 (TP345457)	CAPS, *Bse*DI	227, 39, 12	143, 84, 39, 12	233	24	43

**Fig. 3:**
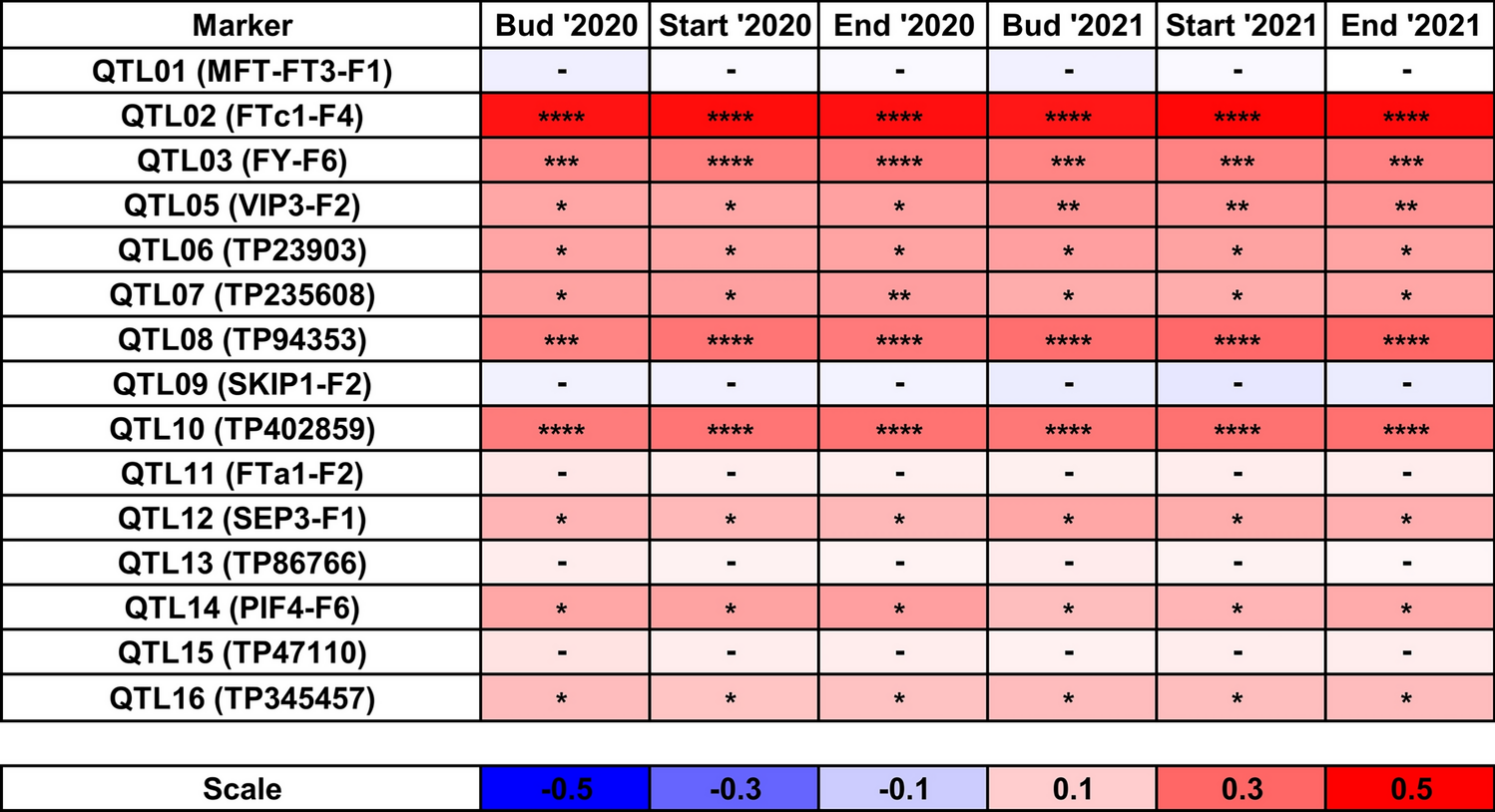
Correlation heatmap reporting Spearman rank correlation coefficients for each trait vs white lupin linkage map QTL PCR marker comparison. Reference alleles were coded as 0, heterozygotes as 1, whereas alternative alleles as 2. Observations were performed during 2020 and 2021 growing seasons in a greenhouse at the Institute of Plant Genetics, Polish Academy of Sciences, Poznań, Poland (52°26′ N 16°54′ E). The bar below the heatmap indicates the color legend of correlation coefficients. Asterisk (*) indicates significant correlations in the following scheme: ****, p < 0.00001; ***, 0.00001 ≤ p < 0.0001; **, 0.0001 ≤ p ≤ 0.001; *, 0.001 ≤ p ≤ 0.05.; –, non-significant.

Taking into consideration correlation with plant phenology, ten QTL markers (Fig. 3) revealed significant correlation (i.e. with a P-value below 0.05), however, only one of these markers, QTL02, had a correlation coefficient value and P-value (mean r value 0.47, P-value 5.3 × 10^–18^) similar to those calculated for the most correlated DArT-seq, silicoDArT and *LalbFTc1* gene INDEL markers. It should be clarified here, that the QTL02 marker is anchored in the *LalbFTc1* gene promoter and amplifies the region carrying markers PR_70 and PR_71a-d. The two other QTL markers highly correlated with plant phenology were QTL08 (mean r value 0.28, P-value 1.2 × 10^–6^) and QTL10 (mean r value 0.27, P-value 1.8 × 10^–6^). Those markers are located in the major flowering time QTLs on chromosomes Lalb_Chr02 and Lalb_Chr13, respectively. Nevertheless, the usefulness of the QTL10 marker for marker-assisted selection may be seriously affected by excessive heterozygosity.

**Fig. 4:**
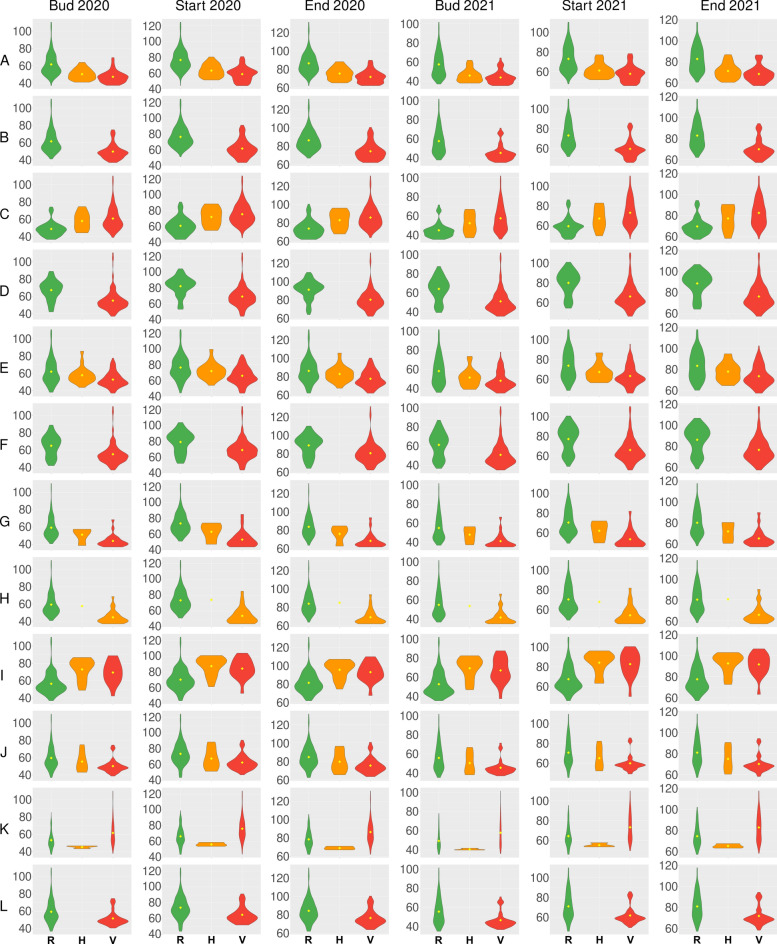
Allelic effects on days from sowing to floral bud emergence (Bud), the start of flowering (Start) and the end of flowering (End) from a 2-year controlled environment study (2020 and 2021) for white lupin markers significantly correlated with plant phenology: Chr16_572706_CAPS (**A**), PR_71d (**B**), QTL02 (**C**), PR_58c (**D**), Chr07_16560064_D_CAPS (**E**), PR_36b (**F**), PR_71b (**G**), PR_42a (**H**), Chr13_1469866_CAPS (**I**), PR_71a (**J**), Chr13_12561729_D_PCR (**K**) and PR_70 (**L**). R stands for a reference allele, V is for a variant allele, whereas H is for a heterozygote. Observations were performed at the Institute of Plant Genetics, Polish Academy of Sciences, Poznań, Poland (52°26′ N 16°54′ E).

To summarize, twelve markers, namely Chr16_572706_CAPS, PR_71d, QTL02, PR_58c, Chr07_16560064_D_CAPS, PR_36b, PR_71b, PR_42a, Chr13_1469866_CAPS, PR_71a and Chr13_12561729_D_PCR, PR_70 revealed strong correlations with plant phenology (P-value < 1 × 10^–7^). These markers also revealed significant correlations (P-value < 1 × 10^–4^) with cumulative growing degree days (GDDs) for all studied traits in both years (Supplementary Tables S8 and S9). As markers differ in allele frequencies (Tables 1, 2, 3) and direction of allelic effects (Fig. 4), they constitute a versatile tool for breeders, enabling the reselection of desired alleles in wide genetic background. In routine screening of the progeny, only a few markers from this panel need to be used, depending on the target phenotype (earliness or vernalization responsiveness) and allelic phases detected in parental lines.

**Fig. 5:**
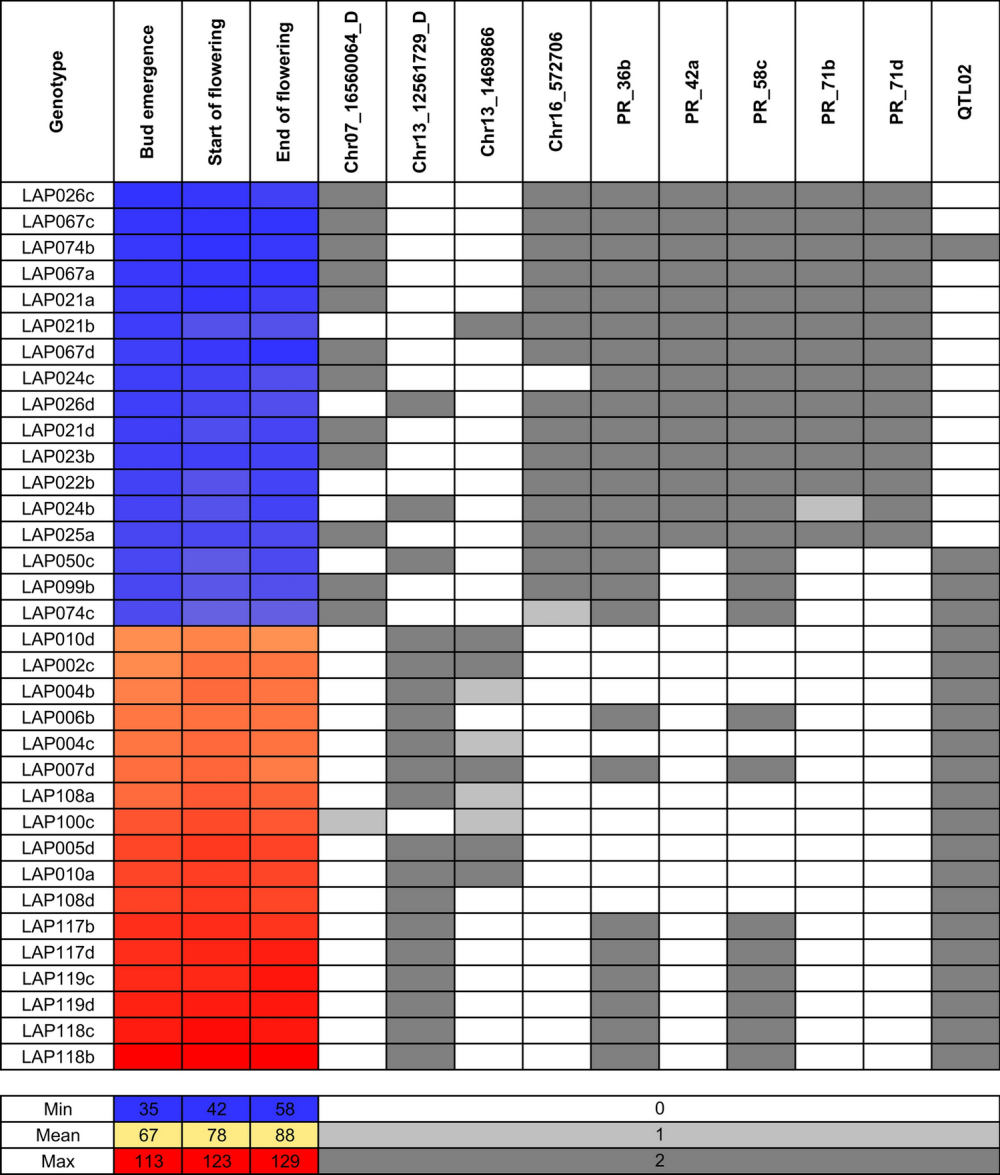
Allelic composition heatmap of PCR markers that revealed the highest correlations with white lupin phenology. Reference alleles were coded as 0, heterozygotes as 1, whereas alternative alleles as 2. Observations were performed during the 2020 and 2021 growing seasons in a greenhouse at the Institute of Plant Genetics, Polish Academy of Sciences, Poznań, Poland (52°26′ N 16°54′ E). The bar below the heatmap indicates the color legend of phenological observations (days from sowing to bud emergence, start of flowering, and end of flowering) and PCR marker alleles. Only genotypes that revealed the most extreme phenology are represented on the heatmap.

Since landraces are dynamic populations that are diverse in their genetic composition both within and between populations, we compared phenotypic variability within and between genotypes of a particular landrace with genetic diversity (Supplementary Table S10). Mean standard deviation values calculated for phenology traits ranged from 1.3 and 1.9 within genotypes and from 3.2 to 3.9 between genotypes (a, b, c, d) within landraces, whereas mean percentages of markers showing different scores between genotypes within landraces ranged from 0.0% to 42.9% for 42 PCR-based markers (mean value of 17.2%) and from 0.8% to 27.7% for 10 720 DArT-seq^46^ markers (mean value of 19.7). There were significant correlations of genetic diversity between PCR-based and DArT-seq markers (0.64, P-value 6.9 × 10^–12^), as well as between genetic diversity of both types of markers and phenotypic diversity (standard deviations) within landraces for all studied phenology traits in both years (from 0.27 to 0.37, P-values from 0.0003 to 0.0065). Therefore, a designed PCR array may also be useful to distinguish one genotype from the others within a particular landrace (Fig. 5).

## Discussion

### The current status of the PCR array for molecular selection of agronomic traits in white lupin

The present study highlighted moderate heritability of time to flowering in the absence of vernalization in white lupin (0.40–0.41). It highlighted quantitative inheritance of this trait in white lupin, contrary to single gene inheritance in the narrow-leafed lupin (*L. angustifolius*) with heritability value about 0.81^53^ and oligogenic inheritance in the yellow lupin (*L. luteus*) with reported heritability about 0.71^54^, whereas other white lupin studies revealed heritability about 0.60–0.64^55,56^. Similar heritability values were also reported in many other legume species, including, inter alia, *Glycine max*^57,58^, *Medicago truncatula*^59,60^, *Vigna unguiculata*^61^ and *Cicer arietinum*^62^. It should be noted that the occurrence of vernalization decreases heritability values for flowering time and this reduction is proportional to the length of vernalization^59^. Therefore, heritability values calculated for field observations with some effective vernalization days are usually lower than those obtained for a controlled environment without any vernalization. Moreover, genetic heterogeneity of landraces may result in underestimation of heritability values due to phenotypic diversity of particular genotypes within a landrace. Our study highlighted relatively high genetic diversity within landraces, resulting in 17–20% polymorphic marker scores between particular genotypes. This finding is coherent with the cross-pollination rate, estimated in white lupin as 10–30%^63,64^.

In theory, moderate and high heritability traits may be effectively scored by traditional phenotyping and marker-assisted selection does not provide large benefits in the means of efficiency^65–67^. Nevertheless, even in such cases, molecular markers may still be very beneficial for the selection of traits that are time-consuming in scoring the phenotypes and/or are environmentally sensitive, such as vernalization responsiveness or disease resistance^68^. Moreover, for the specific traits that are expressed in the adult plants (i.e. induction of flowering) molecular screening and selection of plants can be performed at the juvenile phase of growth, providing an opportunity to reduce the number of lines subjected to further crossings or seed multiplication^69^. The current PCR marker array applicable for white lupin MAS includes several transformed DArT-seq and silicoDArT markers for plant phenology developed and positively validated in this study, several candidate *LalbFTc1* insertion-deletion polymorphism underlying vernalization independence^46^ also positively validated here, a few markers for anthracnose resistance elucidated from linkage mapping study^7,22^, albeit not confirmed in the independently controlled environment and field conditions survey^9^, and a PCR marker tagging a functional mutation (SNP) responsible for the low alkaloid *pauper* phenotype^20,21^. Moreover, there are several candidate SNP loci significantly associated with field-relevant anthracnose resistance awaiting transformation into PCR array^8,70^. Recently, several kompetitive allele-specific PCR (KASP) markers targeting the novel source of white lupin anthracnose resistance have been developed and preliminary validated^71^.

For a closely-related major lupin crop species, *L. angustifolius*, numerous loci linked to agronomic traits have been reported and mapped in the genome, including vernalization responsiveness (*Ku*/*Julius*), pod shattering (*tardus* and *lentus*), soft seededness (*mollis*), low alkaloid content (*iucundus*), white seed color (*leucospermus*), Phomopsis stem blight (*Phr1*, *PhtjR*) and anthracnose resistance (*Lanr1, LanrBo*, *AnMan*)^72–80^. Functional mutations were identified only for vernalization responsiveness (*FLOWERING LOCUS T* gene, *LanFTc1*) and low alkaloid content (*APETALA2*/ethylene response transcription factor, *RAP2-7*)^81–83^. Nevertheless, molecular markers for agronomic trait selection in narrow-leafed lupin breeding were developed for all major traits^78,80,84–99^. Contrary to the statement of^100^ that “the vast majority of the favorable alleles at these identified QTL reside in journals on library shelves rather than in cultivars that have been improved through the introgression or selection of these favorable QTL alleles” all those loci were introduced into narrow-lupin breeding with the great aid of molecular selection^101^. However, it was quite feasible as all of those were single- or double-gene traits. In white lupin, the situation is different because many agronomic traits have quantitative inheritance indicating polygenic control^7–9,23,38,42,43,102–109^. For such traits, traditional selection with a set of PCR-based markers was usually ineffective^110^ and as such has been replaced by genomics-assisted breeding^111,112^.

### Perspectives for genomic selection in white lupin breeding

As the cost of genotyping has significantly decreased, the use of high-density SNP arrays for genomic-enabled selection has become more feasible in modern breeding^113^. During the recent decade, commercially available SNP arrays have been developed for numerous plant species, including cereals, oilseeds, horticultural crops as well as major legumes such as groundnut, chickpea, pigeon pea, cowpea, common bean and soybean^112,114^. New genotyping methods have been consequently introduced into legume breeding and already resulted in the development of improved cultivars^115,116^. Lupin breeding, despite relatively advanced molecular research, was technologically backward as compared to other species, exploiting traditional PCR marker systems for selection, supplemented a few years ago with a Fluidigm nanofluidic array^74,75,77,101^. Just very recently, a multispecies low-cost DNA genotyping platform for chickpeas, field peas, lentils and lupins has been developed by Grains Research Development Corporation and Agriculture Victoria in Australia^117^. The vast majority of mentioned molecular resources are available only for the narrow-leafed lupin, with white lupin lagging behind modern breeding.

Development of reference genome assemblies for the white lupin cultivar Amiga^40,41^ as well as resequencing of a set of modern cultivars, landraces and wild germplasm resources^39^ opened possibilities for GWAS and genomic predictions. Such studies in white lupin have been already performed, targeting anthracnose resistance, drought tolerance, grain yield, yield-related morphological traits, plant height, winter survival and time to flowering^8,9,46,102–104,118^. Depending on the trait and population size reported predictive abilities in those studies ranged from about 0.28 to 0.86.

The cost-effectiveness of genomic selection in plant breeding depends on many factors, including potential genetic gain, the predictive accuracy of the selected model, cost of implementation, and, last but not least, the availability of cost-effective high-throughput SNP arrays or next-generation sequencing platforms^119,120^. Taking into consideration genotyping technology, two platforms were exploited in white lupin: genotyping-by-sequencing with genome complexity reduced with restriction enzyme *Ape*KI^121^ and Diversity Arrays Technology sequencing (DArT-seq)^122^. Both methods are commercially available and provide a large number of markers but are relatively expensive, with a cost per sample of roughly 40–50 USD, whereas newer technology based on liquid chips has reduced genotyping cost to just a few dollars per sample^123–125^. As white lupin is a minor crop species with a low global protein market when compared to other grain legumes, the genotyping cost may be a limiting factor for the implementation of genomic selection into breeding practice. At least in Poland, which in the year 2022 was the second lupin producer in the world, with 193 360 ha of harvested lupin acreage (FAOSTAT, 2024), two major breeding companies working on this species prefer PCR-based genotyping to genomic selection due to financial constraints (personal communication). 

Ongoing climate change may also force reselection of germplasm towards lower vernalization responsiveness due to difficulties in fulfilling vernalization requirements. Central Europe has experienced an increase in air temperature by about 2–2.5 °C since the 1950s in the spring months March-May^126^. For instance, the April-June period in Poland was 2.03 °C warmer in the last decade than in the 1970s, revealing a relatively stable trend of 0.50 ± 0.13 °C per decade^127^. As a consequence of global warming, delayed flowering has already been observed in natural plant populations with high vernalization requirements^128^, as well as in winter wheat in temperate climate, resulting in yield sensitivity to vernalization variations^129^. German wheat breeders compensated for rising temperature in the past recent 50 years by the consecutive release of cultivars with the negative trend for the heading time (about 4 days of earliness improve per decade)^130^. A similar strategy can be adapted for white lupin, with the use of validated markers reported in this study, providing an opportunity for rapid, accurate and effective reselection of early flowering, thermoneutral germplasm in the progeny.

## Materials and methods

### Plant material

The plant material encompassed 300 genotypes: 52 accessions provided by Poznań Plant Breeding Ltd. (Wiatrowo, Poland) and 248 genotypes randomly selected from 120 accessions representing 11 landrace and 2 cultivar pools^43^ provided by Council for Agricultural Research and Economics (Lodi, Italy). Selection of genotypes was performed due to observed high variability of phenology within accessions during 2004–2005 growing season^43^. From two to four genotypes were retained per accession, including early, late and intermediate flowering time. Genotypes originate from 26 countries and differ by domestication status: 243 are landraces, 34 cultivars, 18 wild or primitive accessions and 5 breeding lines. Collection sites represent different climatic conditions such as tropical and subtropical highland (Ethiopia), cold semi-arid (Anatolia and Maghreb), dry-summer Mediterranean (sites around the Mediterranean Basin), warm-summer Mediterranean (Azores and Madeira), humid temperate (i.e. oceanic: French cultivars), temperate subcontinental (other cultivars and breeding lines). These regions diverged also by photoperiod during the juvenile phase of white lupin growth, ranging from about 9–10 h in winter sowing in northern regions of the Mediterranean Basin to 11–12 h in Ethiopia and 12–17 h in spring sowing in other regions of Europe. The list of accessions with countries/regions of origin, domestication status and germplasm donors is provided in Supplementary Table S1.

### Phenotyping of plant phenology in white lupin germplasm panel

Phenotyping of plant phenology was performed in a controlled environment (greenhouse) without pre-sowing vernalization. Experiments were performed at the Institute of Plant Genetics, Polish Academy of Sciences, Poznań, Poland (52°26′ N 16°54′ E) in Poznań (western Poland) with spring sowing (19th March 2020 and 11th March 2021) and under ambient long-day photoperiod, increasing during plant cultivation from about 12 h in March to 16 h in June and July. Automatic heating was used to keep the minimum air temperature above 18 °C. The mean minimum temperature recorded in greenhouse was 18.1 °C in both years, whereas the mean maximum temperature reached 27.1 °C in 2020 and 26.7 °C in 2021. Watering was provided every second day to avoid drought stress. Potting soil was composed of TS1 medium basic rec. 085 substrate pH 6.0 (Klasmann-Deilmann GmbH, Geeste, Germany) mixed with sand in equal proportions. No artificial fertilization was provided during the experiments. Floral bud emergence was scored when the bud was visible after parting the leaves surrounding the apical meristem (inspected every second day). The start of flowering was recorded as the number of days from sowing to the observation of the first fully colored petal on the main stem, whereas the end of flowering was when half of the petals on the main inflorescence faded. Observations were made in at least three biological replicates (depending on the seed availability, from 3 to 10 plants per genotype were analyzed). Experimental design in '2020 and '2021 controlled environment phenotyping of white lupin germplasm panel is provided in Supplementary Table S11. Cumulative growing degree days (GDDs) were calculated using the formula:

$$GDDs = \mathop \sum \limits_{t = 1}^{n} {\text{max}}\left( {Td - Tb;0} \right)$$

where *t* and *n* are days from sowing and the total number of days from sowing to the observed phenology (floral bud emergence, start of flowering and end of flowering), *Td* is a daily mean temperature, whereas *Tb* corresponds to the base temperature of white lupin considered in this study as 3°C^24,28^. GDD values for fractional days were calculated on a linear scale.

Daily mean temperature was calculated using the formula:

$$Td=\frac{Tmax+Tmin}{2}$$

where *Tmax* and *Tmin* are daily maximum and minimum temperatures.

### DNA isolation from white lupin germplasm panel

Two young upper leaves (about 50–100 mg tissue), collected from 5-week-old plants cultivated in a greenhouse, were placed into 2 ml microcentrifuge tubes (Eppendorf, Hamburg, Germany), immediately frozen in liquid nitrogen and stored at − 80 °C. Frozen plant tissue was homogenized for 45 s at 30 rpm using TissueLyser II (Qiagen, Hilden, Germany) and two stainless steel beads (ø 5 mm, Qiagen). DNA isolation was performed with Maxwell® RSC PureFood GMO and Authentication Kit (Promega, Mannheim, Germany)^131^ and automated isolation station Maxwell® RSC 48 Instrument (Promega). No changes to the standard protocol were made. DNA concentration and quality were estimated using a NanoDrop 2000 (ThermoFisher Scientific, Warsaw, Poland). Automated DNA isolation protocol yielded about 95 µl of mixture with an average concentration of 1011 ± 323 ng/ µl DNA (min. 407 ng/µl, max 1998 ng/µl). Results of DNA isolation are provided in Supplementary Table S12. Three biological replicates were analyzed per genotype.

### Development of PCR markers for novel loci associated with flowering time in white lupin germplasm panel

A recently published GWAS highlighted several new loci significantly associated with white lupin flowering time in a range of environments^46^. These loci included two types of Diversity Array Technology (DArT) sequencing data: presence/absence (dominant) markers (SilicoDArT) and standard single nucleotide polymorphism (SNP) markers (DArT-seq). SNP markers were directly transformed into PCR markers using cleaved amplified polymorphic sequence (CAPS)^47^ or derived CAPS (dCAPS)^48^ techniques. SilicoDArT markers required a more complex approach because they may represent several possible types of polymorphisms, including SNPs and small INDELs in restriction enzyme recognition sites, larger insertions/deletions in restriction fragments or methylation variation at restriction sites. Potential sequence polymorphism underlying SilicoDArT markers could be identified by Sanger sequencing, therefore, PCR primers flanking SilicoDArT loci by 200–250 bp in each direction were designed and used for PCR amplification on the DNA templates isolated from lines showing opposite allelic phases. Next, the obtained amplicons were sequenced. Based on recognized polymorphism, PCR presence/absence, CAPS or dCAPS markers were designed. Sanger sequencing was performed using BigDye® Terminator v3.1 Cycle Sequencing Kit (ThermoFisher Scientific) and 96-capillary 3730xl DNA Analyzer (Applied Biosystems, ThermoFisher Scientific) by Genomed (Warsaw, Poland).

Sequence alignments were performed using the progressive Mauve algorithm^132^ assuming genome collinearity. Primers were designed using Primer 3 Plus^133^ implemented in Geneious Prime^134^. Restriction enzymes for the CAPS approach were identified using SNP2CAPs^50^ and current (28.07.2023) REBASE update^51^ whereas restriction enzymes and mismatches in primers for the dCAPS approach were identified using a web-based version of dCAPS Finder 2.0 http://helix.wustl.edu/dcaps/^135^. The list of all designed markers with primer sequences and their coordinates in white lupin genome sequence is provided in Supplementary Table S3. To facilitate germplasm genotyping with new PCR markers, PCR with a gradient of primer annealing temperature in the range between 56 °C and 64 °C was performed first. If amplification efficiency within this range was too low to enable routine screening, a gradient between 50 °C and 58 °C was tested. Primer annealing temperature providing the strongest PCR bands without significant unspecific products or stutter bands was selected.

### Genotyping of white lupin germplasm panel with PCR markers associated with flowering time

The set of PCR markers used for white lupin germplasm genotyping (Supplementary Table S3) included those developed in this study as well as those recently published for QTL loci^42^ and insertion-deletion polymorphism in a promoter region of white lupin *FLOWERING LOCUS T* homolog, *LalbFTc1* gene (*Lalb_Chr14g0364281*)^46^. All PCR reactions were performed using a DNA polymerase GoTaq® Flexi (Promega), a thermal cycler Labcycler Gradient (Sensoquest, Göttingen, Germany), 96-well PCR plates (4titude, Wotton, Surrey, UK) and standard pipet tips (Neptune Scientific, San Diego, USA). Thermo Fisher Scientific and New England Biolabs (Ipswich, USA) were restriction enzyme providers. PCR amplicons and restriction products were resolved by gel electrophoresis using standard (Wide Range, Serva, Heidelberg, Germany) or high-resolution agarose (3:1, Serva) with concentration (1–3%) adjusted according to the size of the expected digestion products. Electrophoresis buffer and gels were prepared using standard TAE: Tris base, acetic acid and EDTA (Serva). To transfer samples between PCR plates and gels, an electronic expandable multichannel pipette (Matrix, Thermo Fisher Scientific) was used. Results of electrophoresis separation were visualized by in-gel SYBRSafe (ThermoFisher Scientific) staining and FastGene FAS-DIGI PRO (Nippon Genetics Europe, Düren, Germany) gel documentation system.

### Data analysis

In the exploration of genetic markers associated with flowering time in white lupin, a statistical framework was employed, utilizing the capabilities of R along with its essential libraries, such as tidyverse and ggplot. The relationship between markers and flowering traits was assessed through Spearman’s rank correlation, with the cor.test function providing a means to determine the significance of these correlations, yielding p-values for the correlation coefficients.

For the analysis of greenhouse data, linear mixed models were pivotal. The model, facilitated by the SpATS package^136^, was articulated as:

$$y=\mu +{\alpha }_{i}+{\beta }_{j}+{\gamma }_{ij}+{g}_{k}+\epsilon$$

where $y$ denotes the response variable (flowering traits), μ represents the overall mean, ${\alpha }_{i}$ and ${\beta }_{j}$ are the random effects for row and column, respectively, ${\gamma }_{ij}$ captures the spatial trend within the greenhouse, and ${g}_{k}$ denotes the genotype effect as a random factor, $\epsilon$ symbolizes the residual error. The spatial trend component was estimated using the SAP function^137^ from the SpATS package enhancing the model’s capacity to accurately reflect spatial variability. Following this, the predict function was utilized to derive breeding values (BLUP) for the response variable, ensuring an accurate estimation of genetic potential across different lines and traits. This approach was methodically applied to each dataset, segmented by year, to ensure a nuanced analysis reflective of annual variations. Heritability was estimated using the generalized broad sense heritability formula:

$${H}^{2}=\frac{{\sum }_{i=s+1}^{m}{k}_{i}}{m-s}$$

where *ki* represents the eigenvalues derived from the genetic effects model, *m* denotes the total number of eigenvalues, and *s* indicates the number of eigenvalues that are zero due to model constraints. This formula is effectively capturing both additive and non-additive genetic variances^138^. This approach offered a comprehensive measure of genetic influence on flowering time in white lupin embodying the analytical rigor and depth of investigation, consistent with the standards of scientific inquiry in elucidating the genetic determinants. To complement the analysis, a violin plot illustrating the distribution of flowering time across lines with different alleles of the analyzed markers was created. This visualization was achieved using the ggplot2 package in R, enhanced by the functionalities of the grid and gridExtra packages.

All methods were carried out in accordance with relevant guidelines and regulations.

## Supplementary Information

Supplementary Information 1.

Supplementary Information 2.

Supplementary Information 3.

Supplementary Information 4.

## Data Availability

All data generated during this study are included in this published article, its Supplementary Information files and in the public repositories as follows: sequences of white lupin PCR-based markers targeting DArT-seq and silicoDArT loci significantly associated with white lupin phenology were deposited in Zenodo under DOI https://doi.org/10.5281/zenodo.10689061 whereas sequence variant data in the European Variation Archive (EVA) at EMBL-EBI (project PRJNA939025, accession number ERZ16297462). Full-length agarose gel electrophoregrams for cropped gel images presented in Supplementary Figure S1 are provided in Supplementary Figure S2.
