# Supplementary material for: Development and validation of PCR marker array for molecular selection towards spring, vernalization-independent and winter, vernalization-responsive ecotypes of white lupin (Lupinus albus L.)
Source: Sci Rep. 2025 Jan 21;15:2659. doi: 10.1038/s41598-025-86482-1 (PMC11751487; doi:10.1038/s41598-025-86482-1)
Supplement: Supplementary file 1 — Supplementary Information 1. [file 41598_2025_86482_MOESM1_ESM.pdf]

Development and validation of PCR marker array for molecular selection towards spring, vernalization-independent and winter, vernalization-responsive ecotypes of white lupin (*Lupinus albus* L.)

Anna Surma, Michał Książkiewicz, Wojciech Bielski, Bartosz Kozak, Renata Galek, Sandra Rychel-Bielska.

Scientific Reports

**The list of Supplementary Materials**

Supplementary Figure S1. Agarose gel electrophoregrams showing polymorphism of PCR-based markers targeting DArT-seq and silicoDArT loci significantly associated with white lupin phenology.

Supplementary Figure S2. Full-length agarose gel electrophoregrams for cropped gel images presented in Supplementary Figure S1.

Supplementary Table S1. The list of white lupin accessions with countries/regions of origin, domestication status and germplasm donors.

Supplementary Table S2. Phenotypic observations recorded in controlled environment for white lupin germplasm panel.

Supplementary Table S3. Mean and standard deviation (SD) values calculated for phenotypic observations recorded in studied environments for white lupin germplasm panel.

Supplementary Table S4. The list of all PCR-based markers used in the study with primer sequences and their coordinates in white lupin genome sequence.

Supplementary Table S5. Results of white lupin germplasm panel genotyping with DArT-seq and silicoDArT PCR-based markers.

Supplementary Table S6. Results of white lupin germplasm panel genotyping with *LalbFTc1* gene promoter INDEL PCR-based markers.

Supplementary Table S7. Results of white lupin germplasm panel genotyping with PCR-based markers for flowering time based on linkage mapping studies.

Supplementary Table S8. Mean growing degree day values (GDDs) calculated for phenotypic observations recorded in studied environments for white lupin germplasm panel.

Supplementary Table S9. Correlations of mean growing degree day values (GDDs) calculated for phenotypic observations recorded in studied environments for white lupin germplasm panel with marker scores.

Supplementary Table S10. Standard deviation (SD) values calculated for phenotypic observations within white lupin genotypes and between genotypes within landraces and correlations with PCR and DArT marker diversity within landraces.

Supplementary Table S11. Experimental design in '2020 and '2021, controlled environment phenotyping of white lupin germplasm panel.

Supplementary Table S12. Results of DNA isolation from white lupin leaf samples using Maxwell® RSC PureFood GMO and Authentication Kit (Promega, Mannheim, Germany) and automated isolation station Maxwell® RSC 48 Instrument (Promega).
