# Supplementary material for: Development and validation of PCR marker array for molecular selection towards spring, vernalization-independent and winter, vernalization-responsive ecotypes of white lupin (Lupinus albus L.)
Source: Sci Rep. 2025 Jan 21;15:2659. doi: 10.1038/s41598-025-86482-1 (PMC11751487; doi:10.1038/s41598-025-86482-1)

Development and validation of PCR marker array for molecular selection towards spring, vernalization-independent and winter, vernalization-responsive ecotypes of white lupin (*Lupinus albus* L.)

Anna Surma, Michał Książkiewicz, Wojciech Bielski, Bartosz Kozak, Renata Galek, Sandra Rychel-Bielska.

Scientific Reports

Supplementary Figure S2. Full-length agarose gel electrophoregrams for cropped gel images presented in Supplementary Figure S1.

Chr02_2625564_D_dCAPS

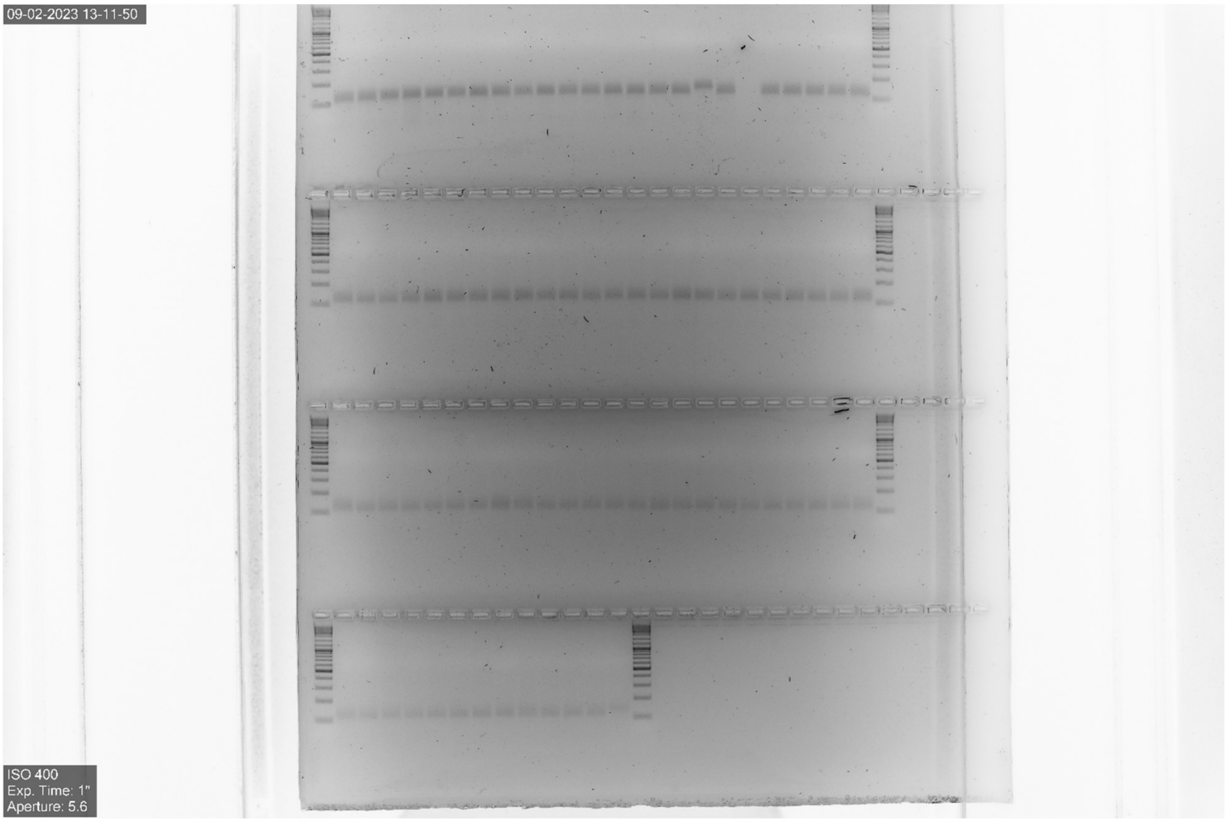

Chr06_14434379_CAPS

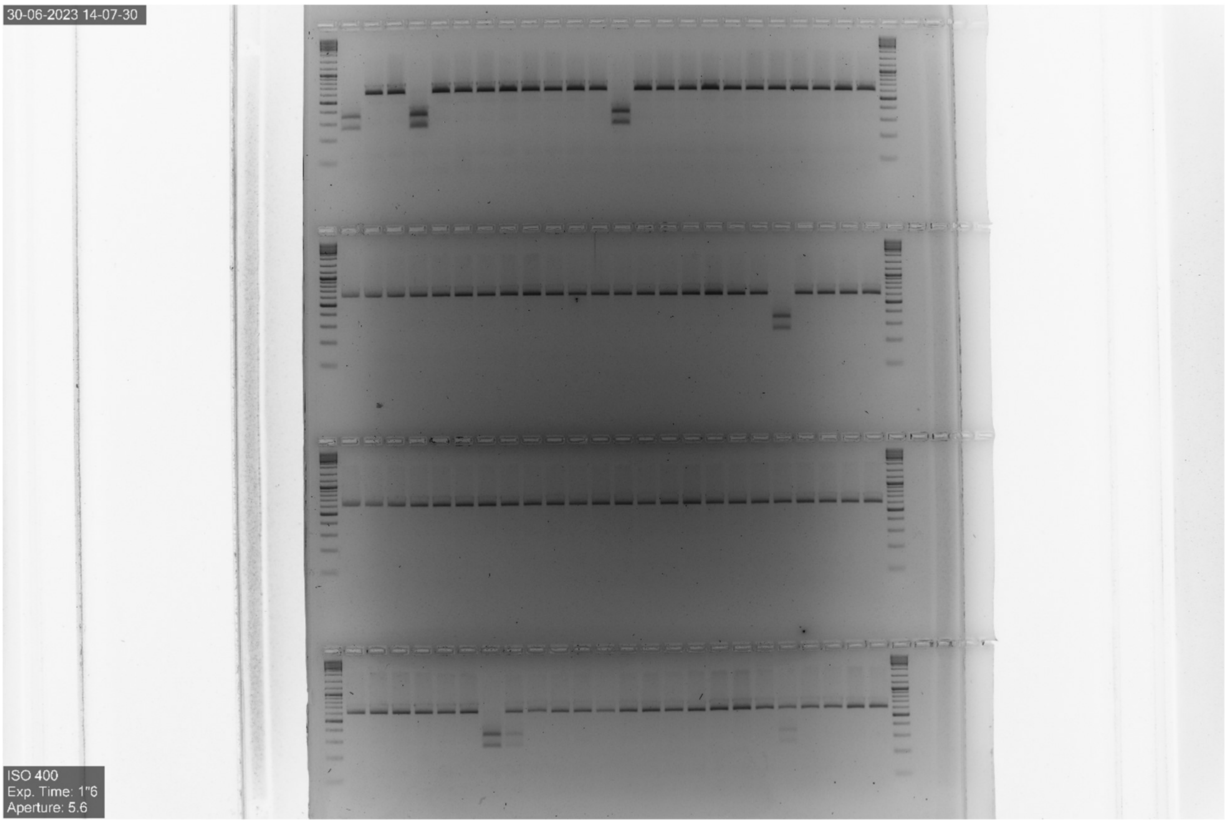

Chr07_16560064_D_CAPS

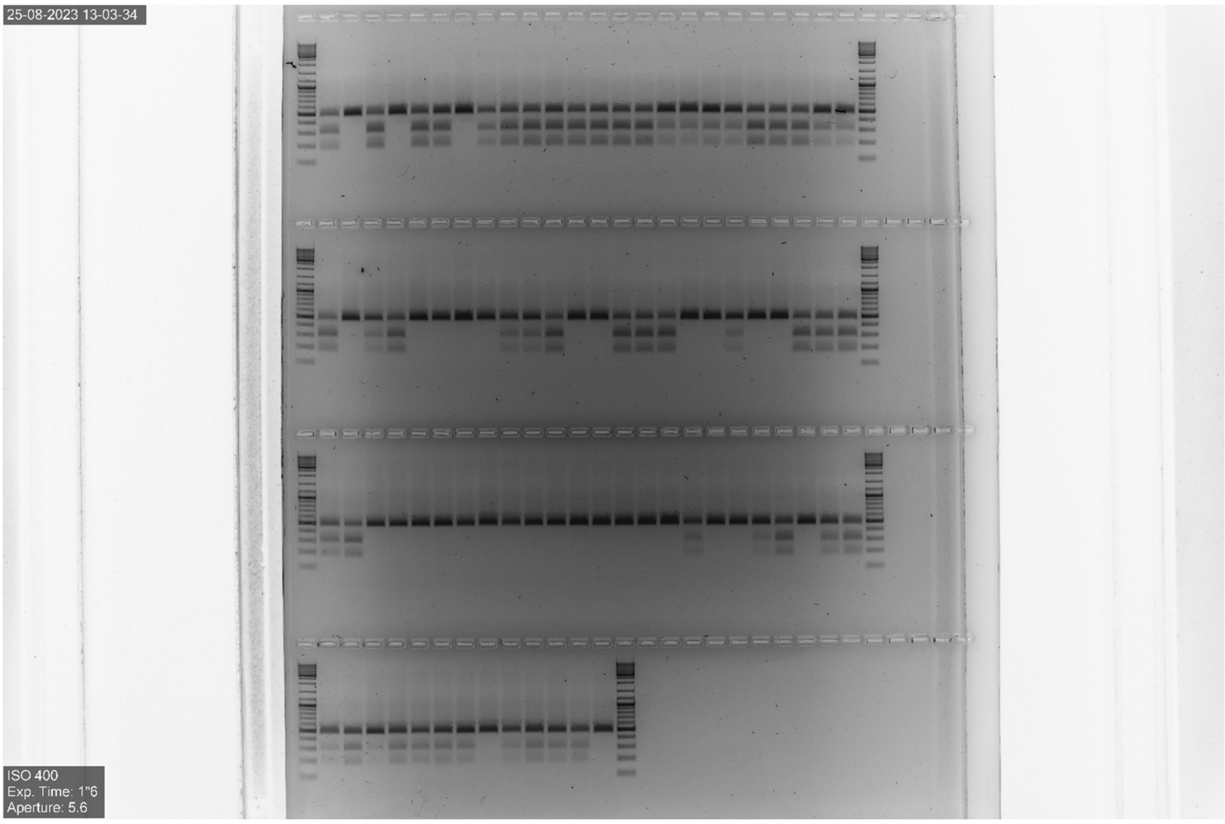

Chr08_12044717_CAPS

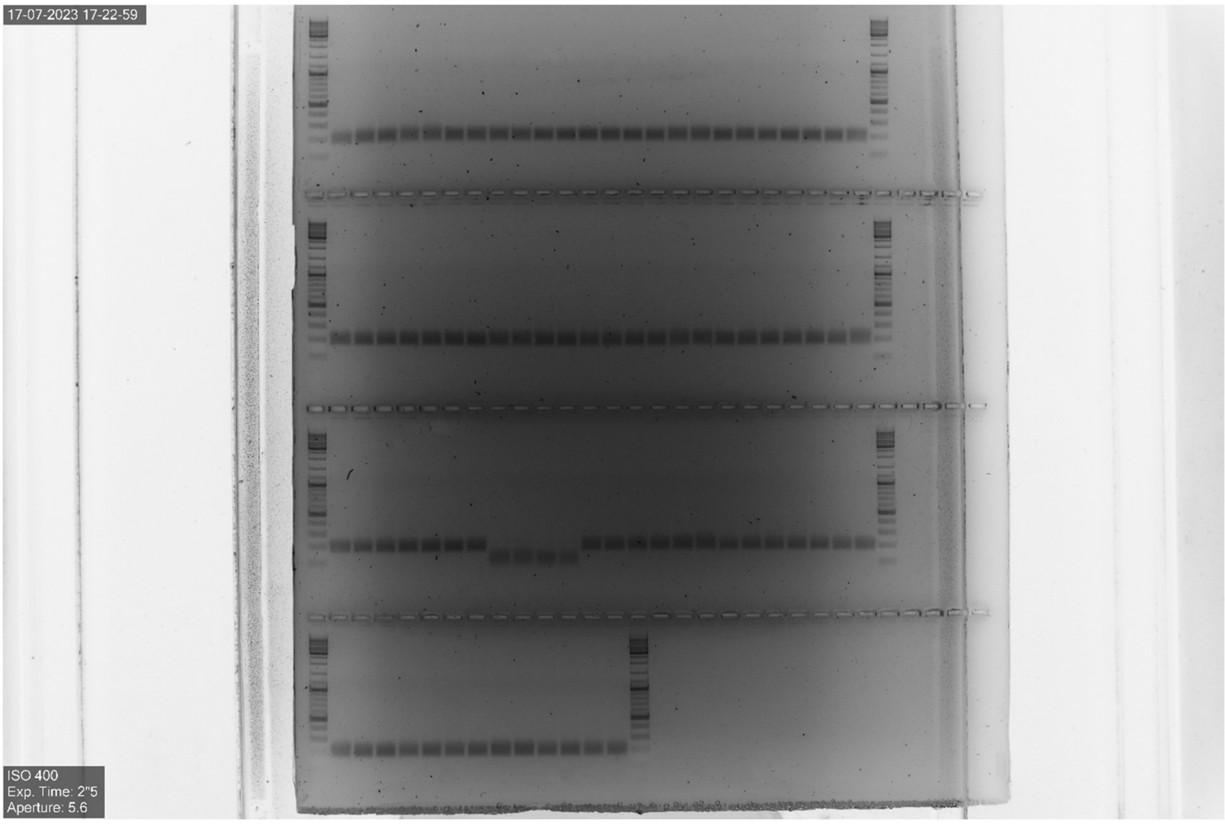

Chr08_3090075_D_dCAPS

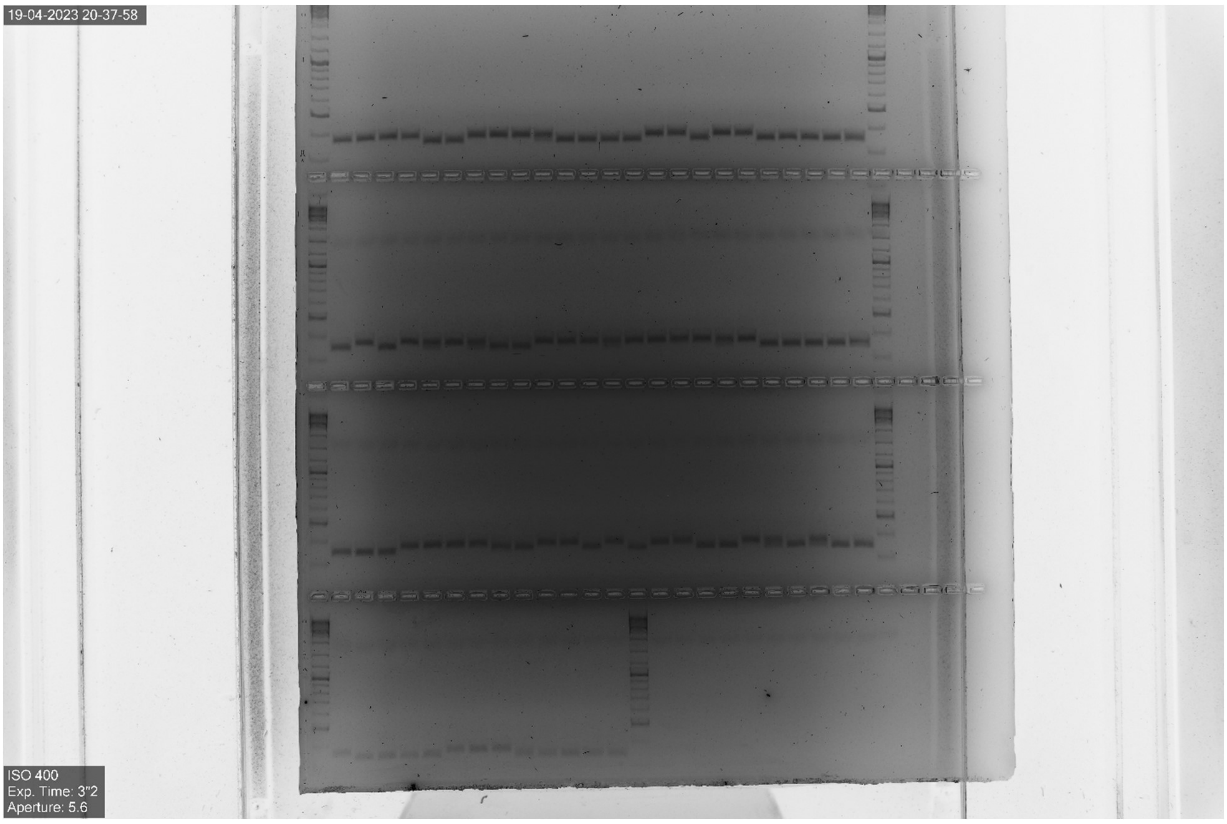

Chr11_14834409_CAPS

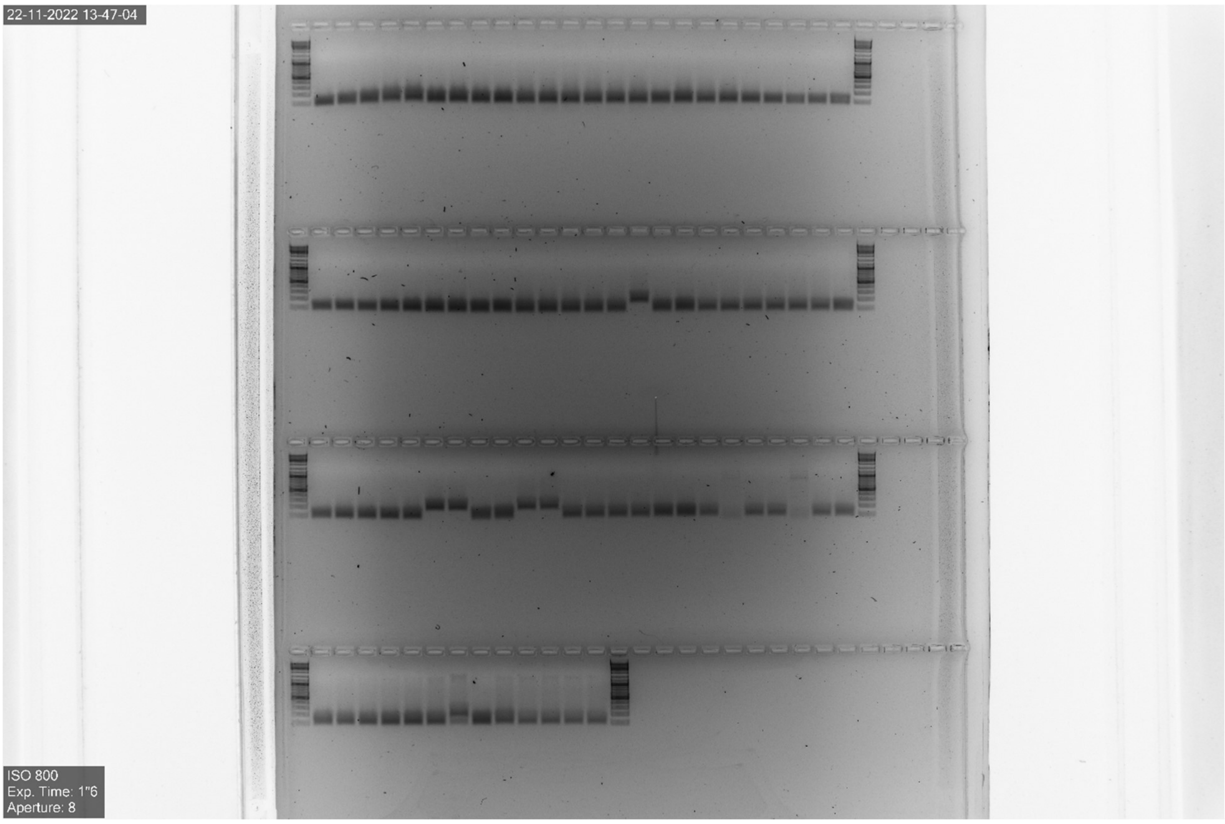

Chr11_5890565_D_dCAPS

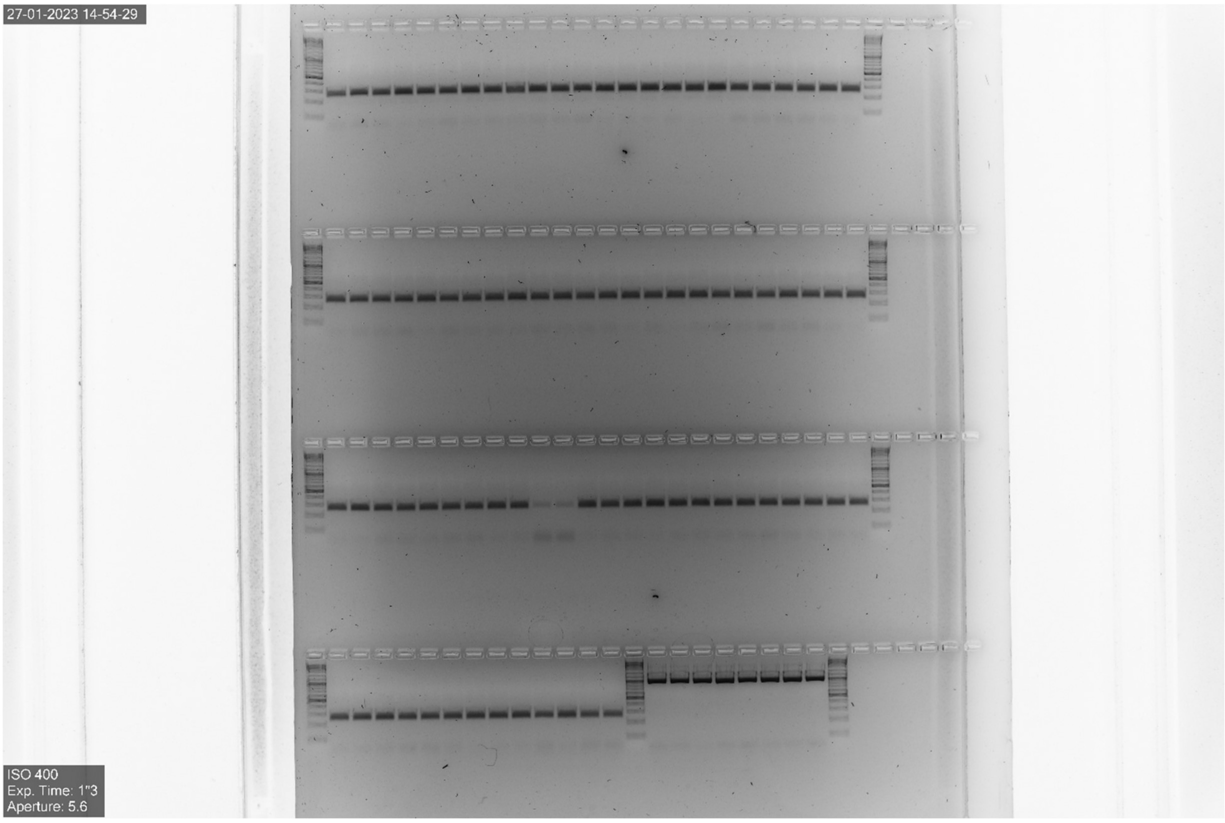

Chr13_12561729_D_PCR

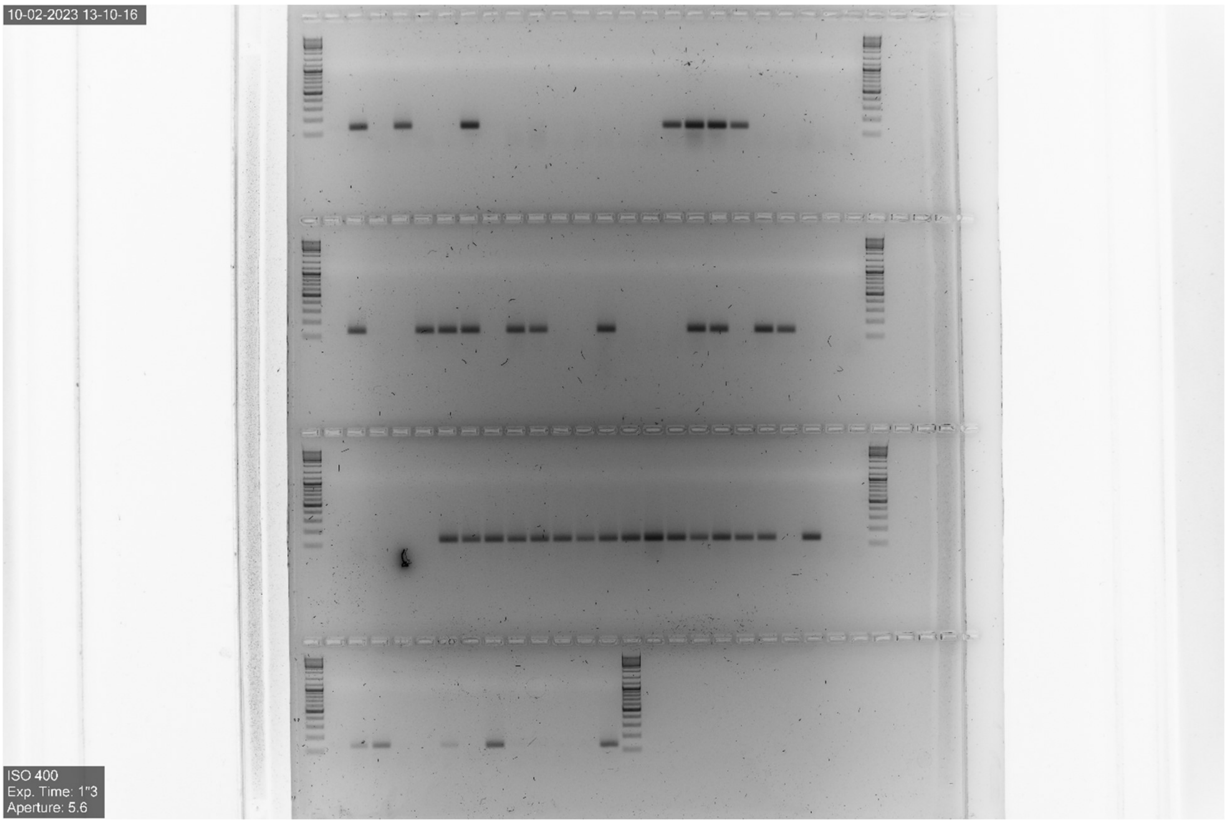

Chr13_1469866_CAPS

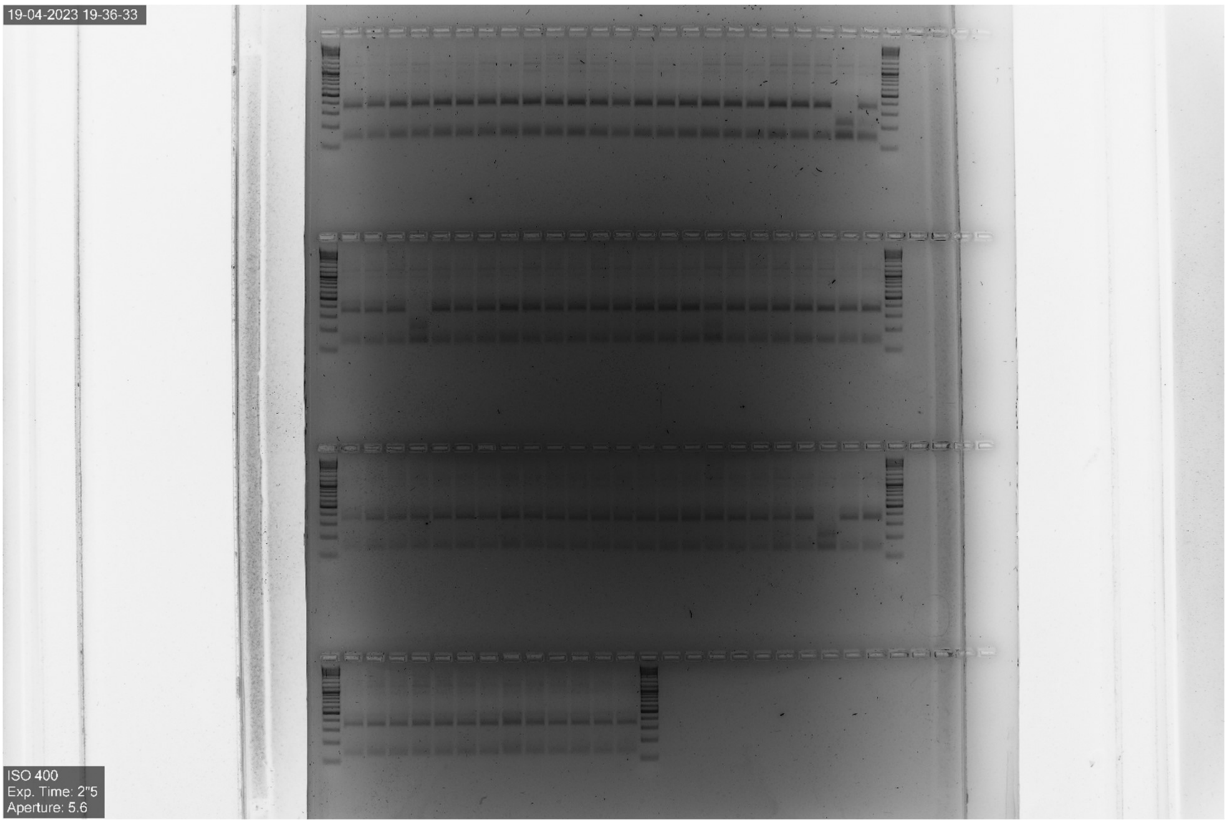

Chr16_572706_CAPS

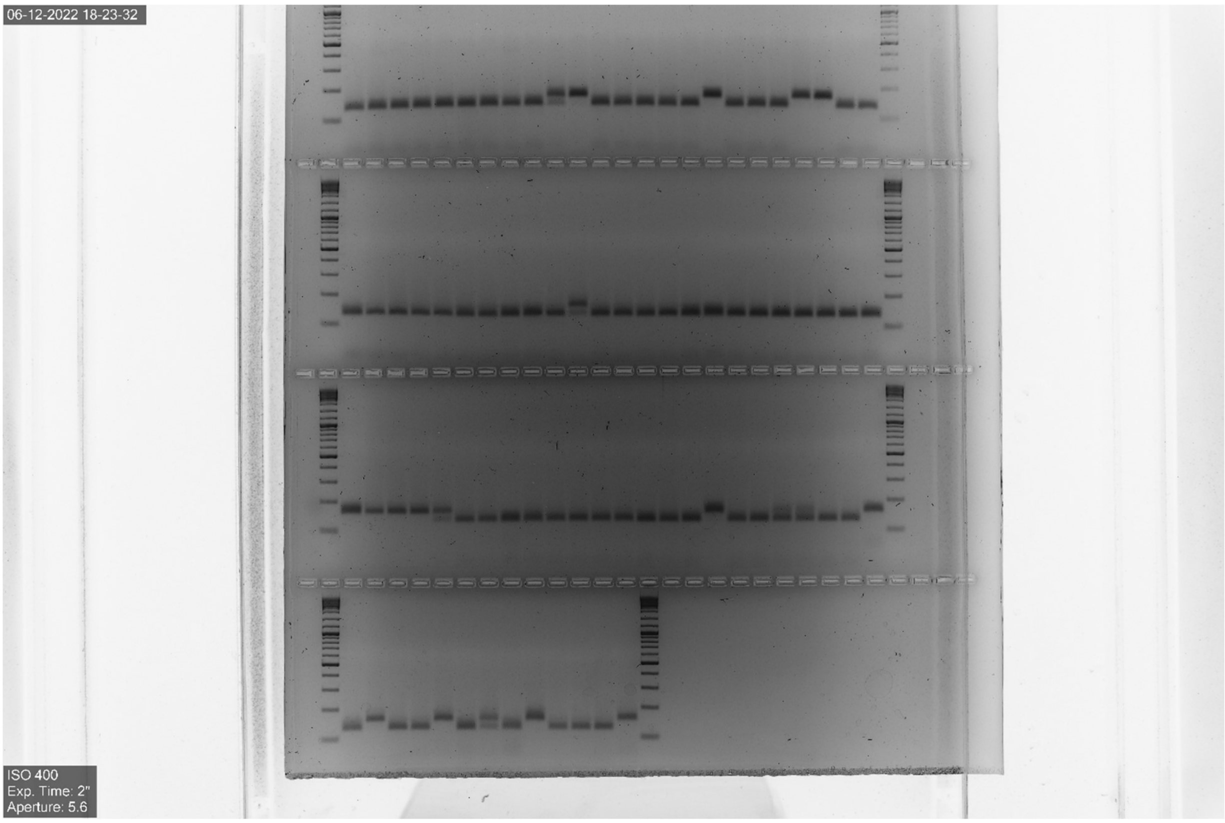

Supplement: Supplementary file 3 — Supplementary Information 3. [file 41598_2025_86482_MOESM3_ESM.pdf]
